# *catena*-Poly[[[diaqua­copper(II)]-bis­[μ-1,1′-(butane-1,4-di­yl)diimidazole-κ^2^ *N* ^3^:*N* ^3′^]] dinitrate]

**DOI:** 10.1107/S1600536808027281

**Published:** 2008-08-30

**Authors:** Xiao-Feng Wang, Jin-Feng Wang, Xiao-Yang Liu

**Affiliations:** a State Key Laboratory of Inorganic Synthesis and Preparative Chemistry, College of Chemistry, Jilin University, Changchun 130012, People’s Republic of China

## Abstract

In the title compound, {[Cu(C_10_H_14_N_4_)_2_(H_2_O)_2_](NO_3_)_2_}_*n*_, the Cu^II^ ion lies on an inversion center and is six-coordinated in an octa­hedral environment by four N atoms from four different 1,1′-butane-1,4-diyldiimidazole ligands and two O atoms from the two water mol­ecules. Bridging by the ligands results in a ribbon structure. Adjacent ribbons are linked to the nitrate anions *via* O—H⋯O hydrogen bonds, forming layers. One nitrate O atom is disordered equally over two positions.

## Related literature

For background and the synthesis of 1,1′-butane-1,4-diyldiimidazole, see: Ma *et al.* (2003 ▶). For the crystal structure of a metal adduct, see: Che *et al.* (2006 ▶).
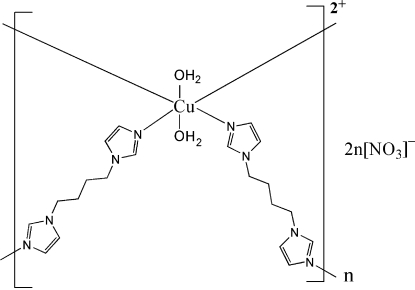


## Experimental

### 

#### Crystal data

[Cu(C_10_H_14_N_4_)_2_(H_2_O)_2_](NO_3_)_2_
*M* *_r_* = 604.10
Monoclinic, 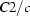

*a* = 22.161 (11) Å
*b* = 10.334 (4) Å
*c* = 14.366 (7) Å
β = 126.375 (18)°
*V* = 2649 (2) Å^3^
*Z* = 4
Mo *K*α radiation
μ = 0.89 mm^−1^
*T* = 291 (2) K
0.48 × 0.36 × 0.25 mm

#### Data collection

Rigaku R-AXIS RAPID diffractometer
Absorption correction: multi-scan (*ABSCOR*; Higashi, 1995 ▶) *T*_min_ = 0.673, *T*_max_ = 0.808
12704 measured reflections
3023 independent reflections
2753 reflections with *I* > 2σ(*I*)
*R*_int_ = 0.024

#### Refinement

*R*[*F* ^2^ > 2σ(*F* ^2^)] = 0.039
*wR*(*F* ^2^) = 0.113
*S* = 1.07
3023 reflections
188 parameters
H-atom parameters constrained
Δρ_max_ = 0.82 e Å^−3^
Δρ_min_ = −0.55 e Å^−3^

### 

Data collection: *RAPID-AUTO* (Rigaku, 1998 ▶); cell refinement: *RAPID-AUTO*; data reduction: *CrystalStructure* (Rigaku/MSC, 2002 ▶); program(s) used to solve structure: *SHELXS97* (Sheldrick, 2008 ▶); program(s) used to refine structure: *SHELXL97* (Sheldrick, 2008 ▶); molecular graphics: *SHELXTL* (Sheldrick, 2008 ▶); software used to prepare material for publication: *SHELXL97*.

## Supplementary Material

Crystal structure: contains datablocks global, I. DOI: 10.1107/S1600536808027281/ng2477sup1.cif

Structure factors: contains datablocks I. DOI: 10.1107/S1600536808027281/ng2477Isup2.hkl

Additional supplementary materials:  crystallographic information; 3D view; checkCIF report

## Figures and Tables

**Table 1:** Hydrogen-bond geometry (Å, °)

*D*—H⋯*A*	*D*—H	H⋯*A*	*D*⋯*A*	*D*—H⋯*A*
O1—H11⋯O4^i^	0.85	1.96	2.801 (4)	170
O5—H12⋯O3^ii^	0.85	2.10	2.888 (8)	153

Symmetry codes: (i) 
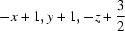
; (ii) 
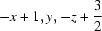
.

**Fig. 1.**
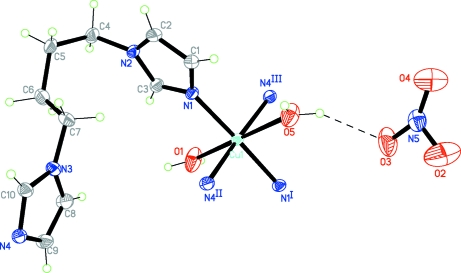
The molecular structure of (I), showing displacement ellipsoids at the 30% probability level for non-H atoms. Dashed lines indicate the hydrogen-bonding interactions [Symmetry code: (I) -x + 1, y, -z + 3/2; (II) -x + 1/2, -y + 3/2, -z - 1;(III) x + 1/2, -y + 3/2, z + 1/2].

**Fig. 2.**
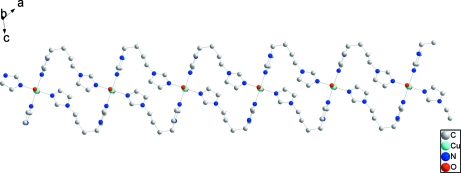
A partial packing view, showing the ribbon chain structure.

**Fig. 3.**
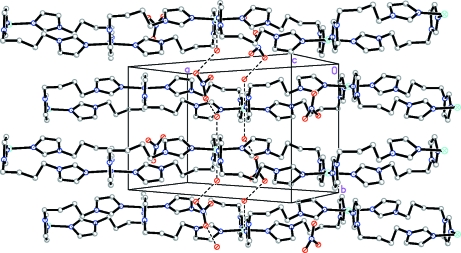
A partial packing view, showing the two-dimensional network. Dashed lines indicate the hydrogen-bonding interactions and H atoms have been omitted.

## supplementary crystallographic information

### Comment

The 1,1'-butane-1,4-diyldiimidazole can be used as a flexible ligand to construct coordination polymeric compounds (Ma *et al.*, 2003; Che *et al.*, 2006). In this paper, we report the new title compound, (I), synthesized by the reaction of 1,1'-butane-1,4-diyldiimidazole ligands and copper dinitrate in methanol.

The Cu^II^ atom is located on an inversion centre and is hexacoordinated by four N atoms of four different 1,1'-butane-1,4-diyldiimidazole ligands and two O atoms of two water molecules (Fig. 1). Adjacent Cu(II) ions are linked by pairs of 1,1'-butane-1,4- diyldiimidazole molecules, resulting in a ribbon motif (Fig. 2).

In the crystal structure, uncoordinated nitrate anions link these ribbons into a layer structure *via* O—H···O hydrogen bonds (Table 1,Figure 3).

### Experimental

1,1'-Butane-1,4-diyldiimidazole was prepared from imidazole and 1,4-dibromobutane in DMSO (Ma *et al.*, 2003). 1,1'-(1,4-Butanediyl)diimidazole (0.380 g, 2 mmol) and copper dinitrate (0.188 g, 2 mmol) were dissolved in hot methanol solution (15 ml) to give a clear solution was obtained. The resulting solution was allowed to stand in a desiccator at room temperature for several days. Blue crystals of (I) were obtained.

### Refinement

The O3 atom of the nitrate is refined with a split model over two positions, with occupancy of 0.5 for O3 and O3'. H atoms bound to C atoms were placed in calculated positions and treated as riding on their parent atoms, with C—H = 0.93 Å (Caromatic); C—H = 0.97 Å (methylene) and with *U*_iso_(H) = 1.2*U*_eq_(C). Water H atoms were initially located in a difference Fourier map, but they were treated as riding on their parent atoms with O—H = 0.85 Å and with *U*_iso_(H) = 1.5*U*_eq_(O).

### Crystal data

**Table:**

[Cu(C_10_H_14_N_4_)_2_(H_2_O)_2_](NO_3_)_2_	*F*_000_ = 1260
*M**_r_* = 604.10	*D*_x_ = 1.515 Mg m^−^^3^
Monoclinic, *C*2/*c*	Mo *K*α radiation λ = 0.71073 Å
Hall symbol: -C 2yc	Cell parameters from 11099 reflections
*a* = 22.161 (11) Å	θ = 3.3–27.5º
*b* = 10.334 (4) Å	µ = 0.89 mm^−^^1^
*c* = 14.366 (7) Å	*T* = 291 (2) K
β = 126.375 (18)º	Block, blue
*V* = 2649 (2) Å^3^	0.48 × 0.36 × 0.25 mm
*Z* = 4	

### Data collection

**Table:**

Rigaku R-AXIS RAPID diffractometer	3023 independent reflections
Radiation source: fine-focus sealed tube	2753 reflections with *I* > 2σ(*I*)
Monochromator: graphite	*R*_int_ = 0.024
*T* = 291(2) K	θ_max_ = 27.5º
ω scans	θ_min_ = 3.3º
Absorption correction: Multi-scan(ABSCOR; Higashi, 1995)	*h* = −28→28
*T*_min_ = 0.673, *T*_max_ = 0.808	*k* = −13→12
12704 measured reflections	*l* = −18→18

### Refinement

**Table:**

Refinement on *F*^2^	Secondary atom site location: difference Fourier map
Least-squares matrix: full	Hydrogen site location: inferred from neighbouring sites
*R*[*F*^2^ > 2σ(*F*^2^)] = 0.039	H-atom parameters constrained
*wR*(*F*^2^) = 0.113	*w* = 1/[σ^2^(*F*_o_^2^) + (0.0644*P*)^2^ + 3.5066*P*] where *P* = (*F*_o_^2^ + 2*F*_c_^2^)/3
*S* = 1.07	(Δ/σ)_max_ = 0.001
3023 reflections	Δρ_max_ = 0.82 e Å^−^^3^
188 parameters	Δρ_min_ = −0.55 e Å^−^^3^
Primary atom site location: structure-invariant direct methods	Extinction correction: none

### Special details

**Table:**

Geometry. All e.s.d.'s (except the e.s.d. in the dihedral angle between two l.s. planes) are estimated using the full covariance matrix. The cell e.s.d.'s are taken into account individually in the estimation of e.s.d.'s in distances, angles and torsion angles; correlations between e.s.d.'s in cell parameters are only used when they are defined by crystal symmetry. An approximate (isotropic) treatment of cell e.s.d.'s is used for estimating e.s.d.'s involving l.s. planes.
Refinement. Refinement of *F*^2^ against ALL reflections. The weighted *R*-factor *wR* and goodness of fit *S* are based on *F*^2^, conventional *R*-factors *R* are based on *F*, with *F* set to zero for negative *F*^2^. The threshold expression of *F*^2^ > σ(*F*^2^) is used only for calculating *R*-factors(gt) *etc*. and is not relevant to the choice of reflections for refinement. *R*-factors based on *F*^2^ are statistically about twice as large as those based on *F*, and *R*- factors based on ALL data will be even larger.

### Fractional atomic coordinates and isotropic or equivalent isotropic displacement parameters (Å^2^)

**Table:**

	*x*	*y*	*z*	*U*_iso_*/*U*_eq_	Occ. (<1)
C1	0.43494 (12)	0.5667 (2)	0.87357 (19)	0.0360 (4)	
H1	0.4387	0.4802	0.8600	0.043*	
C2	0.40800 (13)	0.6108 (2)	0.9310 (2)	0.0395 (5)	
H2	0.3901	0.5611	0.9638	0.047*	
C3	0.44106 (11)	0.7749 (2)	0.87476 (18)	0.0321 (4)	
H3	0.4495	0.8593	0.8627	0.038*	
C4	0.38590 (14)	0.8327 (3)	0.9787 (2)	0.0460 (6)	
H4A	0.4039	0.9190	0.9807	0.055*	
H4B	0.4069	0.8078	1.0576	0.055*	
C5	0.30100 (14)	0.8355 (3)	0.9086 (2)	0.0514 (7)	
H5A	0.2872	0.8926	0.9467	0.062*	
H5B	0.2836	0.7493	0.9084	0.062*	
C6	0.26042 (14)	0.8803 (3)	0.7830 (2)	0.0473 (6)	
H6A	0.2128	0.9186	0.7569	0.057*	
H6B	0.2902	0.9470	0.7804	0.057*	
C7	0.24623 (12)	0.7730 (2)	0.70083 (18)	0.0394 (5)	
H7A	0.2938	0.7405	0.7214	0.047*	
H7B	0.2205	0.7023	0.7082	0.047*	
C8	0.22282 (12)	0.9038 (3)	0.5330 (2)	0.0427 (5)	
H8	0.2696	0.9427	0.5699	0.051*	
C9	0.16308 (12)	0.9201 (3)	0.42203 (19)	0.0402 (5)	
H9	0.1619	0.9731	0.3687	0.048*	
C10	0.12939 (11)	0.7874 (2)	0.49805 (18)	0.0325 (4)	
H10	0.1010	0.7315	0.5086	0.039*	
Cu1	0.5000	0.66433 (3)	0.7500	0.02769 (13)	
N1	0.45588 (9)	0.67011 (16)	0.83845 (14)	0.0293 (4)	
N2	0.41221 (9)	0.74259 (19)	0.93120 (14)	0.0328 (4)	
N3	0.20075 (10)	0.81828 (18)	0.58036 (16)	0.0342 (4)	
N4	0.10424 (9)	0.84658 (17)	0.39970 (15)	0.0317 (4)	
N5	0.59796 (14)	0.1501 (2)	0.7259 (3)	0.0516 (6)	
O1	0.5000	0.9027 (3)	0.7500	0.0740 (10)	
H11	0.4628	0.9523	0.7254	0.111*	
O2	0.5825 (2)	0.0978 (4)	0.6366 (3)	0.1229 (13)	
O3	0.5894 (5)	0.2662 (7)	0.7011 (8)	0.089 (2)	0.50
O4	0.61485 (19)	0.0723 (3)	0.8025 (3)	0.0997 (10)	
O5	0.5000	0.4120 (3)	0.7500	0.0640 (8)	
H12	0.4690	0.3573	0.7433	0.082 (13)*	
O3'	0.6140 (5)	0.2592 (7)	0.7753 (9)	0.099 (3)	0.50

### Atomic displacement parameters (Å^2^)

**Table:**

	*U*^11^	*U*^22^	*U*^33^	*U*^12^	*U*^13^	*U*^23^
C1	0.0384 (11)	0.0388 (11)	0.0349 (10)	0.0060 (9)	0.0239 (9)	0.0065 (9)
C2	0.0400 (11)	0.0502 (13)	0.0353 (11)	0.0056 (10)	0.0263 (10)	0.0109 (10)
C3	0.0270 (9)	0.0405 (11)	0.0284 (9)	−0.0014 (8)	0.0162 (8)	−0.0031 (8)
C4	0.0390 (12)	0.0706 (17)	0.0274 (11)	0.0129 (11)	0.0191 (10)	−0.0063 (10)
C5	0.0388 (12)	0.089 (2)	0.0311 (12)	0.0203 (12)	0.0233 (11)	0.0022 (11)
C6	0.0419 (12)	0.0596 (14)	0.0324 (11)	0.0185 (11)	0.0178 (10)	0.0020 (11)
C7	0.0314 (10)	0.0485 (13)	0.0256 (10)	0.0068 (9)	0.0099 (9)	0.0054 (9)
C8	0.0284 (10)	0.0633 (15)	0.0339 (11)	−0.0104 (10)	0.0170 (9)	−0.0013 (10)
C9	0.0313 (10)	0.0588 (14)	0.0309 (10)	−0.0083 (10)	0.0187 (9)	0.0017 (10)
C10	0.0263 (9)	0.0397 (10)	0.0281 (10)	−0.0016 (8)	0.0144 (8)	0.0021 (8)
Cu1	0.02036 (18)	0.0421 (2)	0.02152 (19)	0.000	0.01289 (15)	0.000
N1	0.0239 (8)	0.0408 (9)	0.0241 (8)	0.0015 (6)	0.0147 (7)	−0.0004 (6)
N2	0.0254 (8)	0.0509 (10)	0.0216 (8)	0.0055 (7)	0.0135 (7)	−0.0009 (7)
N3	0.0252 (8)	0.0453 (10)	0.0258 (9)	0.0011 (7)	0.0116 (7)	0.0020 (7)
N4	0.0249 (8)	0.0436 (9)	0.0260 (8)	−0.0019 (7)	0.0147 (7)	0.0002 (7)
N5	0.0477 (12)	0.0460 (12)	0.0739 (17)	0.0010 (9)	0.0430 (13)	0.0065 (11)
O1	0.098 (2)	0.0383 (14)	0.134 (3)	0.000	0.095 (3)	0.000
O2	0.162 (3)	0.137 (3)	0.090 (2)	−0.032 (3)	0.086 (3)	−0.006 (2)
O3	0.112 (6)	0.045 (3)	0.150 (7)	0.023 (4)	0.098 (6)	0.024 (5)
O4	0.109 (2)	0.108 (2)	0.0686 (16)	−0.0290 (19)	0.0449 (16)	0.0012 (16)
O5	0.086 (2)	0.0417 (14)	0.097 (2)	0.000	0.072 (2)	0.000
O3'	0.085 (5)	0.043 (3)	0.158 (8)	0.002 (3)	0.066 (6)	0.001 (5)

### Geometric parameters (Å, °)

**Table:**

C1—C2	1.352 (3)	C8—C9	1.349 (3)
C1—N1	1.374 (3)	C8—N3	1.370 (3)
C1—H1	0.9300	C8—H8	0.9300
C2—N2	1.365 (3)	C9—N4	1.372 (3)
C2—H2	0.9300	C9—H9	0.9300
C3—N1	1.325 (3)	C10—N4	1.322 (3)
C3—N2	1.339 (3)	C10—N3	1.335 (3)
C3—H3	0.9300	C10—H10	0.9300
C4—N2	1.465 (3)	Cu1—N1	2.0120 (18)
C4—C5	1.519 (4)	Cu1—N1^i^	2.0120 (18)
C4—H4A	0.9700	Cu1—N4^ii^	2.020 (2)
C4—H4B	0.9700	Cu1—N4^iii^	2.020 (2)
C5—C6	1.535 (3)	Cu1—O1	2.463 (3)
C5—H5A	0.9700	Cu1—O5	2.608 (3)
C5—H5B	0.9700	N4—Cu1^ii^	2.0203 (19)
C6—C7	1.510 (3)	N5—O4	1.228 (4)
C6—H6A	0.9700	N5—O3	1.234 (7)
C6—H6B	0.9700	N5—O2	1.239 (4)
C7—N3	1.470 (3)	O1—H11	0.8500
C7—H7A	0.9700	O5—H12	0.8500
C7—H7B	0.9700		
			
C2—C1—N1	109.2 (2)	C8—C9—H9	125.1
C2—C1—H1	125.4	N4—C9—H9	125.1
N1—C1—H1	125.4	N4—C10—N3	111.30 (19)
C1—C2—N2	106.46 (19)	N4—C10—H10	124.3
C1—C2—H2	126.8	N3—C10—H10	124.3
N2—C2—H2	126.8	N1—Cu1—N1^i^	176.60 (10)
N1—C3—N2	110.72 (19)	N1—Cu1—N4^ii^	89.82 (8)
N1—C3—H3	124.6	N1^i^—Cu1—N4^ii^	90.37 (8)
N2—C3—H3	124.6	N1—Cu1—N4^iii^	90.37 (8)
N2—C4—C5	112.4 (2)	N1^i^—Cu1—N4^iii^	89.82 (8)
N2—C4—H4A	109.1	N4^ii^—Cu1—N4^iii^	173.60 (10)
C5—C4—H4A	109.1	N1—Cu1—O1	88.30 (5)
N2—C4—H4B	109.1	N1^i^—Cu1—O1	88.30 (5)
C5—C4—H4B	109.1	N4^ii^—Cu1—O1	93.20 (5)
H4A—C4—H4B	107.8	N4^iii^—Cu1—O1	93.20 (5)
C4—C5—C6	114.7 (2)	N1—Cu1—O5	91.70 (5)
C4—C5—H5A	108.6	N1^i^—Cu1—O5	91.70 (5)
C6—C5—H5A	108.6	N4^ii^—Cu1—O5	86.80 (5)
C4—C5—H5B	108.6	N4^iii^—Cu1—O5	86.80 (5)
C6—C5—H5B	108.6	O1—Cu1—O5	180.000 (1)
H5A—C5—H5B	107.6	C3—N1—C1	105.91 (18)
C7—C6—C5	113.8 (2)	C3—N1—Cu1	126.85 (14)
C7—C6—H6A	108.8	C1—N1—Cu1	127.24 (14)
C5—C6—H6A	108.8	C3—N2—C2	107.66 (18)
C7—C6—H6B	108.8	C3—N2—C4	126.1 (2)
C5—C6—H6B	108.8	C2—N2—C4	126.2 (2)
H6A—C6—H6B	107.7	C10—N3—C8	107.36 (18)
N3—C7—C6	111.5 (2)	C10—N3—C7	126.27 (19)
N3—C7—H7A	109.3	C8—N3—C7	126.33 (19)
C6—C7—H7A	109.3	C10—N4—C9	105.44 (18)
N3—C7—H7B	109.3	C10—N4—Cu1^ii^	127.10 (15)
C6—C7—H7B	109.3	C9—N4—Cu1^ii^	127.43 (15)
H7A—C7—H7B	108.0	O4—N5—O3	144.0 (5)
C9—C8—N3	106.16 (19)	O4—N5—O2	113.1 (3)
C9—C8—H8	126.9	O3—N5—O2	103.0 (5)
N3—C8—H8	126.9	Cu1—O1—H11	127.1
C8—C9—N4	109.7 (2)	Cu1—O5—H12	131.6

Symmetry codes: (i) −*x*+1, *y*, −*z*+3/2; (ii) −*x*+1/2, −*y*+3/2, −*z*+1; (iii) *x*+1/2, −*y*+3/2, *z*+1/2.

### Hydrogen-bond geometry (Å, °)

**Table:**

*D*—H···*A*	*D*—H	H···*A*	*D*···*A*	*D*—H···*A*
O1—H11···O4^iv^	0.85	1.96	2.801 (4)	170
O5—H12···O3^i^	0.85	2.10	2.888 (8)	153

Symmetry codes: (iv) −*x*+1, *y*+1, −*z*+3/2; (i) −*x*+1, *y*, −*z*+3/2.
